# Chemsex and its repercussions on the health of men who have sex with men (MSM): a global health perspective

**DOI:** 10.1590/0034-7167-2023-0004

**Published:** 2023-08-07

**Authors:** Alvaro Francisco Lopes de Sousa, Emerson Lucas Silva Camargo, Isabel Amélia Costa Mendes

**Affiliations:** I Hospital Sírio-Libânes, Instituto de Ensino e Pesquisa. São Paulo, São Paulo, Brazil; II Universidade Nova de Lisboa, Instituto de Higiene e Medicina Tropical. Lisboa, Portugal; III Universidade de São Paulo. Ribeirão Preto, São Paulo, Brazil

**Keywords:** Chemsex, Men, Men’s Health, Sexually Transmitted Infections, Global Health, Chemsex, Hombres, Salud del Hombre, Enfermedades de Transmisión Sexual, Salud Global, Chemsex, Homens, Saúde do Homem, Infecções Sexualmente Transmissíveis, Saúde Global

## Abstract

**Objectives:**

to discuss the repercussions of chemsex on the health of men who have sex with men (MSM), contextualizing it in a global health scenario and pointing out the implications for nursing care.

**Methods:**

theoretical-reflexive study based on scientific literature and concepts related to global health.

**Results:**

we present the epidemiology of the chemsex phenomenon, the main demands of the field, the reasons why it has become a global public health problem, and the implications for nursing practice.

**Final Considerations:**

chemsex is growing in all age groups of MSM and is globally benefiting from location-based applications to gain magnitude, finding an important potential audience in the migrant population. Nursing structures can help accelerate the proposal and implementation of biomedical and behavioral measures to address chemsex in its entirety, qualifying care and inducing teamwork with interprofessional collaboration.

## RESUMO

**Objetivos:**

discutir as repercussões do *chemsex* na saúde de homens que fazem sexo com homens (HSH), contextualizando-a num cenário de saúde global e apontando as implicações para os cuidados da enfermagem.

**Métodos:**

estudo teórico-reflexivo fundamentado na literatura cientifica e nos conceitos relacionados a saúde global.

**Resultados:**

apresentamos a epidemiologia do fenômeno *chemsex* , as principais demandas do campo, os motivos pelo qual tornou-se um problema de saúde pública global e as implicações para a atuação da enfermagem.

**Considerações Finais:**

o *chemsex* vem crescendo em todos os segmentos etários de HSH e globalmente vem se beneficiando dos aplicativos baseado em geolocalização para ganhar magnitude, encontrando na população migrante, um importante público em potencial. Estruturas de enfermagem podem ajudar a acelerar a propor e implementar globalmente medidas biomédicas e comportamentais de enfrentamento ao *chemsex* em sua totalidade, que qualifiquem o cuidado e induzam ao trabalho em equipe com colaboração interprofissional.

## RESUMEN

**Objetivos:**

discutir las repercusiones de *chemsex* en la salud de hombres que tienen sexo con hombres (HSH), contextualizándolas en un escenario de salud global y señalando las implicaciones para los cuidados de enfermería.

**Métodos:**

estudio teórico-reflexivo fundamentado en la literatura científica y en los conceptos relacionados con la salud global.

**Resultados:**

presentamos la epidemiología del fenómeno *chemsex* , las principales demandas del campo, los motivos por los cuales se ha convertido en un problema de salud pública global y las implicaciones para la actuación de enfermería.

**Consideraciones Finales:**

el *chemsex* está aumentando en todos los segmentos de edad de HSH y globalmente está beneficiándose de aplicaciones basadas en la geolocalización para aumentar su magnitud, encontrando en la población migrante un importante público potencial. Las estructuras de enfermería pueden ayudar a acelerar la propuesta e implementación global de medidas biomédicas y conductuales para abordar el *chemsex* en su totalidad, que califiquen el cuidado e induzcan al trabajo en equipo con colaboración interprofesional.

## INTRODUCTION

The term “chemsex” is a contraction of “chemical sex” used to indicate the voluntary ingestion of psychoactive and non-psychoactive drugs in the context of sexual parties and sexual relationships. It is particularly associated with the population of men who have sex with men (MSM) to facilitate prolonged sexual sessions, almost always associated with multiple sexual partners^( 1 )^.

This population may have significant and multifaceted relationships with drugs. Research in England indicates that the rate of alcohol dependence among MSM is twice that of the non-MSM male population and that gay and bisexual men are three times more likely to use illicit substances than their heterosexual peers^( 2 )^. Studies conducted in Brazil indicate that among MSM, the prevalence of chemsex is high, ranging from 27% to 69.9%^( 3 , 4 )^ depending on which drugs are considered, a trend also observed in Portugal, where the prevalence ranges from 9% to 30%^( 5 )^.

The main difficulty in providing a global overview of the problem is that there is still no universally accepted definition of which drugs make up the “chemsex phenomenon”^( 6 , 7 )^. Generally, it includes licit and illicit drugs capable of altering the subjects’ perception such as alcohol; opioids (such as heroin, codeine, and other synthetic substances); cannabinoids (marijuana, hashish, synthetic cannabinoids, spices); sedatives or hypnotics (barbiturates, benzodiazepines); cocaine; stimulants (such as amphetamines); hallucinogens (LSD, ecstasy), sex-performance-enhancing drugs (poppers), and others^( 5 )^.

Due to these characteristics, chemsex is commonly associated with exposure to sexually transmitted infections (STIs), since the situation of sex mediated by the use of psychoactive substances is commonly associated with risky sexual behaviors such as unprotected anal sex, with unknown partners, exchange of sexual partners during group sex, dryness, dehydration, and loss of sensitivity, increasing the chances of injuries and bleeding^( 8 )^.

Abscesses, lacerations, and other problems in the anal region are common in studies with chemsex practitioners, as the effects of drugs include analgesia facilitating fisting, footing, double penetration, and other challenging sexual practices. In addition, the difficulty of reasoning can often decrease the ability and willingness to use condoms and correctly use protective measures such as pre-exposure prophylaxis (PrEP) or post-exposure prophylaxis (PEP)^( 6 , 7 , 8 )^.

This is a highly complex phenomenon with several associated risks, but it continues to be addressed on a case-by-case basis in scientific literature and in the formulation of public policies. As the largest healthcare workforce, the participation of nursing in this discussion is of utmost importance. Advanced nursing structures can help accelerate the proposal and globally implement biomedical and behavioral measures to address chemsex in its entirety, which qualify care and induce teamwork with interprofessional collaboration. For this, nursing care delivery models should be based on the acquisition of additional skills through practical experience, developing an expanded scope of practice over the years. Additionally, it should focus on principles of interprofessional collaborative practice that sustain ethical and professional nursing care standards to promote safe transitions and effective divisions in care for chemsex practitioners.

## OBJECTIVES

To discuss the repercussions of chemsex on the health of men who have sex with men, contextualizing it in a global health scenario and pointing out the implications for nursing care.

## METHODS

This is a theoretical-reflexive study aligned with the vision and experience of researchers in the theme of global health, determinants of the health of vulnerable populations, human exposome and population mobility, based on the researchers’ work in two international research networks: the Global Health and Tropical Medicine (GHTM) and the Human Exposome and Infectious Diseases Network (HEID).

The main points of discussion were derived from reflective analysis supported by the authors’ production on the subject and the most current scientific literature. In order to discuss the implications for nursing care, we sought to present the epidemiology of the chemsex phenomenon in the population of MSM, the reason why it has become a global public health problem, and the implications for nursing practice.

## DISCUSSION

### The epidemiology of chemsex

There are significant variations in the estimates of prevalence for chemsex globally in the literature. A systematic review study^( 9 )^ identified a prevalence ranging from 4% to 94%, while another study^( 6 )^ reported an overall prevalence of 17% (CI: 3% to 29%). Additionally, the two reviews identified that the drugs used in chemsex were also used outside of sexual events, causing mental distress and worsening the individuals’ quality of life. The results of both studies indicate that the prevalence of chemsex varies between countries but also within different regions and cities of the same country, mainly according to geographic location^( 6 , 9 )^.

The type of drug to be considered also influences the estimates of prevalence. For example, research involving methamphetamines is more “antiquated” and common in Europe, North America, and Australia. This can be explained by the high value of this drug, as well as the fact that the MSM population has a longer history of using this drug. On the other hand, research examining GHB is more common in Western Europe and is more recent^( 6 , 7 , 8 , 9 )^.

In Brazil, little is studied about chemsex. A study^( 4 )^ that compared the prevalence of this phenomenon in Brazil during the COVID-19 pandemic, combining a series of drugs, had an overall prevalence of 38.9%. European countries with which Brazil has a great commercial and immigrant relationship, Portugal also registers a lack of studies involving chemsex in MSM, although a 2021 study points to an overall prevalence of 20.2%^( 5 )^, also in the context of COVID-19. There is also the problem of overlapping vulnerabilities and generations, which affects older MSM who practice chemsex.

A study conducted in Italy and the United Kingdom, with men living with HIV over 45 years of age who practiced chemsex, indicated that the research participants classify their involvement with chemsex as driven by the pursuit of sociability combined with a rediscovery of sexual pleasure and an enhanced sense of comfort with their bodies resulting from the emergence of the HIV undetect-ability paradigm^( 10 )^. However, the drug also causes experimentation with insecurities and discomfort resulting from the combination of drugs, duration of the effect, and side and late effects.

Since chemsex is a socially constructed phenomenon, the use of specific drugs varies among different cultures and subpopulations of MSM^( 9 )^, but common factors such as language can facilitate the perpetuation of this behavior. This limits the generalization of the findings, which is reflected in the different studies that consist of very varied types of samples. The socially constructed nature of chemsex explains the variation in estimates of prevalence and types of drugs used in different geographic areas but reinforces the need for comparative studies that allow understanding the phenomenon as a global health problem.

### The environment for the diffusion of chemsex practices

Combined drug use during sex is not a new phenomenon, but it appears to have been amplified and facilitated by new technologies for interaction and relationships, such as geolocation-based applications. The exacerbating factor of these apps lies in their ability to provide individuals with an “escape” to be “themselves” without exposing the truth about their preferences, fetishes, and sexual behaviors^( 11 )^. However, there is a gap in understanding the impact that new forms of relationship using social media can have on the practice of chemsex: connecting new partners with common objectives, drug use in social contexts, and providing increasingly easier ways to find psychoactive substances, all anonymously^( 4 , 5 , 11 )^.

Grindr and Hornet are the two most popular dating apps for gay and bisexual male users^( 12 )^. These apps use the GPS in cell phones to indicate nearby contacts, usually in busy areas of major cities^( 4 , 12 )^. Identifying sympathizers and suppliers of illicit drugs is relatively easy on these platforms, as there is a “standard and international” nomenclature based on emojis that facilitate contact. For example, the lightning bolt ( 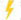 ) and snowflake ( 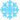 ) indicate cocaine use, in Brazil, Portugal, the United States or any country that uses these apps. There are several other emojis used to standardize the meaning of drugs, such as the use of explosion ( 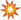 ) to represent alkyl nitrites or isopropyl nitrites, commonly known as poppers, while diamonds ( 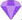 ) and rings ( 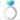 ) are used to represent methamphetamines. The key ( 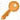 ) is also highlighted, representing “key” (for ketamine, a potent horse anesthetic), and the water droplet ( 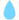 ) for “gi or gisele,” representing GHB or gamma-hydroxybutyrate, another potent anesthetic.

However, abbreviations are also commonly used, such as GHB for gamma-hydroxybutyrate, TK for cocaine, and MD for methamphetamine. Unlike other apps commonly used by the general population, such as Tinder, these apps do not offer user search filters or confirm user identity, facilitating drug sales, exchanges, and purchases on the apps, particularly by international migrants^( 4 , 13 , 14 )^, who seek socialization spaces and groups upon arriving in new countries. However, data on chemsex patterns among immigrants are even scarcer. It is known that traditionally, moving to a new country tends to expose individuals to situations that enhance the adoption of sexual behaviors with greater exposure to STIs, into which chemsex fits.

Although knowledge about these drugs appears to be benefiting from a simple, “universal” language that is easily understood and propagated, the same cannot be assumed about knowledge regarding their use to reduce harm. Partnership with apps can be extremely useful in this case, providing a direct communication channel with this population.

### Nursing care for chemsex participants

Advanced nursing structures can help accelerate the global proposal and implementation of biomedical and behavioral measures to address *chemsex* in its entirety, qualifying care and promoting interprofessional collaboration. However, there are many challenges to be faced in achieving this goal.

A Dutch study^( 1 )^ conducted with nurses showed that almost all (97%) of the participating IST specialists who work in a reference clinic in the country have addressed *chemsex* during their regular consultations with MSM clients. Most of these nurses report regularly addressing *chemsex* due to the information provided by the client. Furthermore, although the majority of nurses have received some training in the area of *chemsex* , 20% of them indicated that their knowledge was focused only on STIs with a deficit in drug ingestion methods, harm reduction measures, and protocols for referral to health institutions. For these reasons, professionals reported having insufficient connection with MSM *chemsex* practitioners and reported a need for more advanced training, which is why they did not feel fully prepared to holistically address their clients^( 1 , 11 )^.

It should be part of the nurse’s practice to address *chemsex* , identify risks, provide information, signal problematic use, and refer clients to other health organizations when necessary and within the logic of care networks^( 1 , 15 )^. For decades, nursing professionals have improved the health of vulnerable populations through a unique and holistic approach that emphasizes community health, health education, and prevention and social well-being. Health education is already a hallmark of nursing care and the basis for many highly effective interventions that support patient self-management in promotion and prevention practices^( 16 , 17 , 18 )^. Health education is the means by which the client’s knowledge is enhanced - a fundamental step in activating motivational progress towards healthy behavior.

Regarding the deficits identified by nurses as limiting their practice and comprehensive care focused on all the needs of MSM *chemsex* practitioners, it is understood that these can be better addressed when based on interprofessional and collaborative practice. A single disciplinary care perspective is insufficient when considering the human being in its entirety, which in itself invites diverse knowledge to dialogue.

In this sense, collaborative practice considers “shared care among healthcare professionals who work in an integrated, interprofessional manner, with coordination of actions, technical and scientific knowledge, and common goals focused on the needs of users”^( 19 )^. In collaborative practice, dialogue with other knowledge is essential because the person receiving care, who does not belong to a single discipline, is at the forefront of the process. In this approach, professionals learn from each other to promote improvements in health outcomes, maximizing the strengths and skills of each category/professional, and contributing to the reduction of isolated, duplicated, and ineffective actions. On the other hand, effective actions are strengthened through shared decision-making, which prioritizes the client, family, or community^( 20 )^.

Furthermore, it is important to propose evidence-based interventions capable of generating changes in the behaviors of this highly vulnerable group, with implications for public and global health aligned with the priorities of the global development agenda, especially regarding Sustainable Development Goals (SDGs) 3: Good Health and Well-being and 10: Reduced Inequalities^( 21 )^.

Agenda 2030 and SDG 3 established target 3.5 to “strengthen the prevention and treatment of substance abuse, including drug abuse and harmful use of alcohol”; as well as to end the AIDS epidemic by 2030, launching ambitious plans focused on testing; early treatment; and viral suppression in those already in treatment, especially in more vulnerable groups, such as MSM. However, this strategy has difficulty fully addressing all groups and subgroups, focusing on immigrants, especially those who are irregular or undocumented, who may find themselves in a situation of great social and programmatic vulnerability in new countries^(^22^)^. Also, due to the important focus of SDG 10 on the contemporary challenge of migrations and flows of displaced people between countries and regions, we understand that greater attention to the problem of *chemsex* can also contribute to achieving the objectives of these two SDGs, giving leadership to Nursing in the global scenario.

## FINAL CONSIDERATIONS

*Chemsex* is a common practice among men who have sex with men (MSM) and is associated with engagement in risky and challenging sexual practices, sexually transmitted infections, chemical dependence, deterioration in quality of life, among others. Although drug use in the context of chemsex is typically done in a sexual setting, its potential effects are not restricted to these settings and can impact human needs across multiple dimensions, requiring a comprehensive, user-centered approach. Nursing professionals should strive to provide safe, competent, and ethical care to those engaging in chemsex, operating within an environment that promotes an interprofessional perspective and collaboration among healthcare professionals for the benefit of the user.
